# Retraction Note to: Differential modulatory effects of GSK-3b and HDM2 on sorafenib-induced AIF nuclear translocation (programmed necrosis) in melanoma

**DOI:** 10.1186/s12943-021-01378-8

**Published:** 2021-06-15

**Authors:** Qingjun Liu, James W. Mier, David J. Panka

**Affiliations:** 1 Division of Hematology-Oncology, Beth Israel Deaconess Medical Center and Harvard Medical School, Boston, MA USA; 2 Division of Urology, Beijing Friendship Hospital, Capital Medical University, Beijing, China

**Retraction Note to: Mol Cancer 10, 115 (2011)**

**https://doi.org/10.1186/1476-4598-10-115**

The Editor-in-Chief has retracted this article after an investigation by Harvard Medical School and Beth Israel Deaconess Medical Center and additional analysis by the Office of Research Integrity found:

- Reuse and relabelling of the same source bands to falsely represent different experimental results in Figure 3A (first four lanes in the middle row of the left panel representing c-myc expression in nucleus of A375 cell type, and first four lanes in the middle row of the right panel representing c-myc expression in nucleus of SK MEL 5 cell type)
- Reuse and relabelling of the same source bands to falsely represent different experimental results in Figure 5 (sixth to eighth lanes of the middle row representing BCL-XL expression in A375-GSK cells with DOX, and last three lanes of the middle row representing BCL-XL expression in SK MEL 5 S9A)
- Reuse and relabelling of unrelated source bands falsely represent different experimental results in Figure 5 (thirteenth to fifteenth lanes of the top row representing BCL2 expression in SK MEL 5 S9A, and thirteenth to fifteenth lanes of the bottom row representing VINCULIN expression in SK MEL 5 S9A)

James W Mier agrees with this retraction. David J Panka does not agree with this retraction. The Editor-in-Chief has not been able to find a current email address for Qingjun Liu.

